# Tapering in patients with chronic pain and prescription opioid use

**DOI:** 10.1192/j.eurpsy.2025.96

**Published:** 2025-08-26

**Authors:** A. Schellekens

**Affiliations:** Psychiatry, Radboudumc, Nijmegen, Netherlands

## Abstract

**Abstract:**

Opioid use disorder in patients with chronic pain poses a specific clincal challenge. Tapering opioids can initially increase pain, while continuing opioids is associated with tolerance and opioid induced hyperalgesia, resulting in inadequate analgesia. In the long run tapering of opioids or rotation to long-acting alternatives, such as buprenoprphine or methadone, have been associated with less pain and better qualtity of life. In this presentation, evidence for various tapering and rotation strategies will be presented, as well as the possiblities for integrated pain management and addiction care. After this session you will know how to prepare your patient with presciroption opioid use disorder and chronic pain for tapering of opioids, how to support your patient with tapering, including the pace of tapering and the use of supproting pharmacological and non-pharmacoligcal interventions. Finally, it will be discussed who is more likely to benefit from rotation to long-acting opioids, and why some patients might be better of without opioid tapering.

**Disclosure of Interest:**

A. Schellekens Grant / Research support from: PI in several studies, funded by national governmental funding agencies.

